# Subconjunctival Acute Bilateral Hemorrhages Due to Kawasaki Disease in a Costa Rican Girl: An Unusual Clinical Manifestation of the Disease

**DOI:** 10.7759/cureus.10212

**Published:** 2020-09-02

**Authors:** Jiulliana Montenegro-Villalobos, Brian Miranda-Jiménez, María L Ávila-Aguero, Rolando Ulloa-Gutierrez

**Affiliations:** 1 Pediatric Infectious Diseases, Hospital Nacional de Niños "Dr. Carlos Sáenz Herrera", San Jose, CRI; 2 Pediatric Medicine, Hospital de San Carlos, Alajuela, CRI; 3 Pediatric Infectious Diseases, Hospital Nacional de Niños "Dr. Carlos Sáenz Herrera", San José, CRI; 4 Pediatric Infectious Diseases, Center for Infectious Disease Modeling and Analysis, Yale School of Public Health, New Haven, USA

**Keywords:** ocular manifestations, subconjunctival hemorrhages, uveitis, kawasaki disease

## Abstract

Kawasaki disease is an acute systemic vasculitis and is the leading cause of acquired cardiac disease in children. Among the ocular manifestations in these patients, bilateral non-suppurative conjunctival injection and uveitis are the most common. We describe a six-year-old Costa Rican girl with acute Kawasaki disease who developed severe bilateral conjunctival injection with subsequent bilateral subconjunctival hemorrhages. For her ocular involvement, she was treated expectantly, and after six weeks there was complete resolution. To our knowledge, this is the first report from Latin America and among the few in the literature of a child in whom severe bilateral subconjunctival hemorrhages occur as a manifestation of Kawasaki disease.

## Introduction

Kawasaki disease (KD) is an acute systemic vasculitis and the leading cause of acquired cardiac disease in children, with approximately 80% of cases occurring in children in the first five years of age. Among ocular manifestations in these patients, non-suppurative conjunctivitis and acute uveitis are the most common and both are usually self-limited [1]. This report describes the presence of bilateral subconjunctival hemorrhages as an unusual manifestation of KD in a girl who was transferred to the only national pediatric tertiary referral and academic hospital of Costa Rica, which belongs to the network of hospitals of the Caja Costarricense de Seguro Social (CCSS). Maternal written informed consent and institutional authorization were obtained for the publication of this report.

## Case presentation

**Figure 1:**
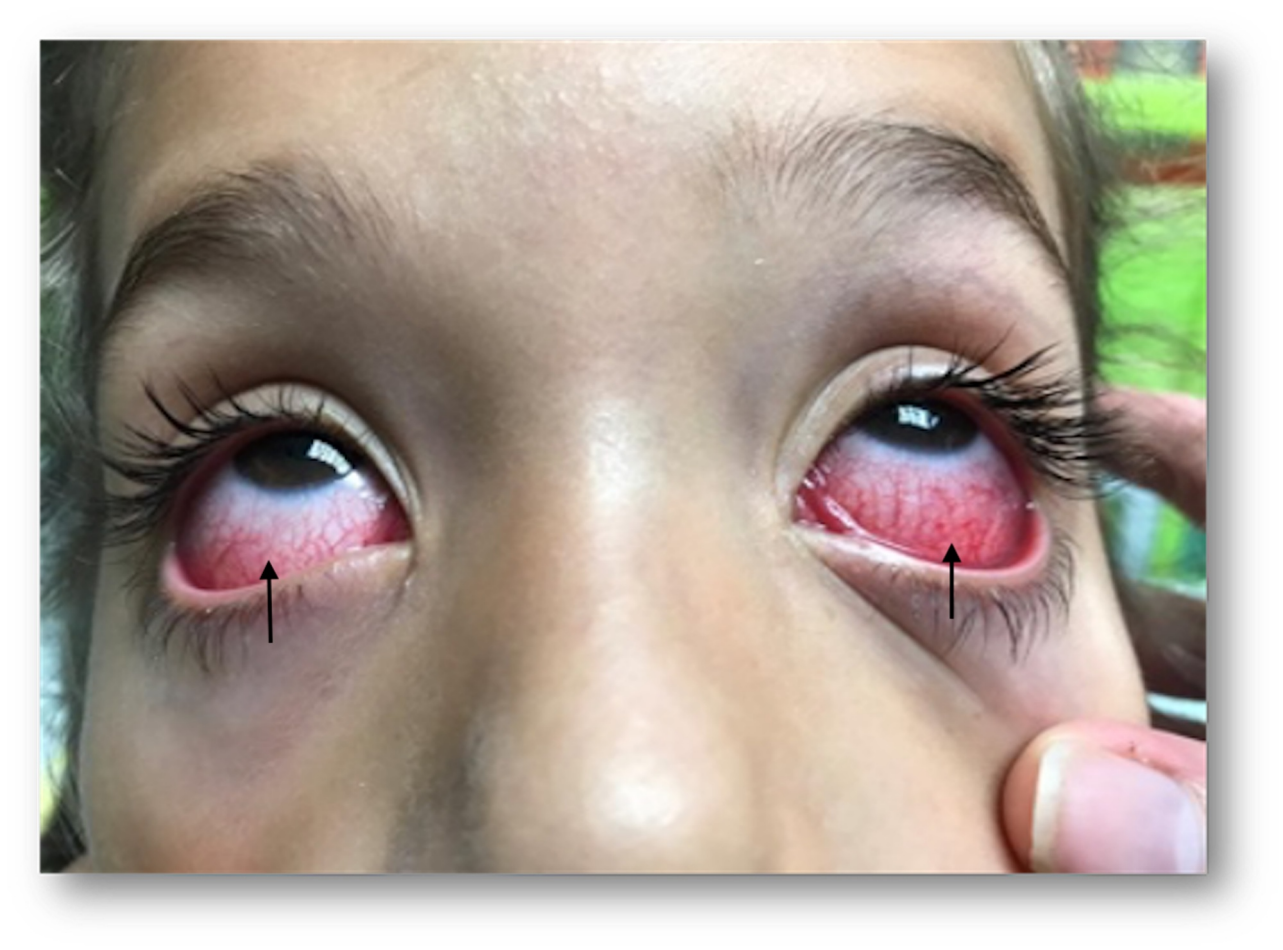
Bilateral subconjunctival hemorrhage with limbar sparing at initial presentation.

A six-year-old girl presented to a regional peripheral hospital with a six-day history of persistent fever, bilateral non-suppurative bulbar conjunctivitis, cheilitis, a non-tender cervical right-side 1.5 cm diameter lymphadenopathy, and diffuse abdominal pain. She had been treated during the last three days with oral amoxicillin/clavulanic acid for an upper respiratory infection but had no improvement. Acute KD was clinically suspected because of persistent fever despite antibiotic and antipyretic treatment, oral mucositis, and strawberry tongue, and the girl was referred to our center for specialized management and treatment. Upon direct admission to our pediatric infectious disease ward, temperature was 38.8°C, heart rate 118/min, blood pressure 113/73 (mean arterial pressure [MAP] 86) mmHg, pulse oximetry 94% 02 saturation. Non-suppurative bilateral conjunctivitis with limbar sparing (Figure 1) was documented, strawberry tongue, and right-side non-tender cervical lymphadenopathy, and looked acutely ill. Laboratory findings showed complete blood count hemoglobin 12.7 g/dL, hematocrit 34.8%, 6770 leukocytes/mm3 (neutrophils 2300, 2480 lymphocytes, 30 eosinophils), and 285,000 platelets/mm3. C-reactive protein (CPR) was 59 mg/dL (normal range <20 mg/dL), and biochemistry tests were normal. No sterile pyuria was documented. Intravenous immunoglobulin (IVIG) at 2 g/Kg on single infusion, and oral acetylsalicylic acid (ASA) at 80 mg/kg/d were initiated. The patient evolved satisfactorily with fever resolution over the next 24 hours.

**Figure 2:**
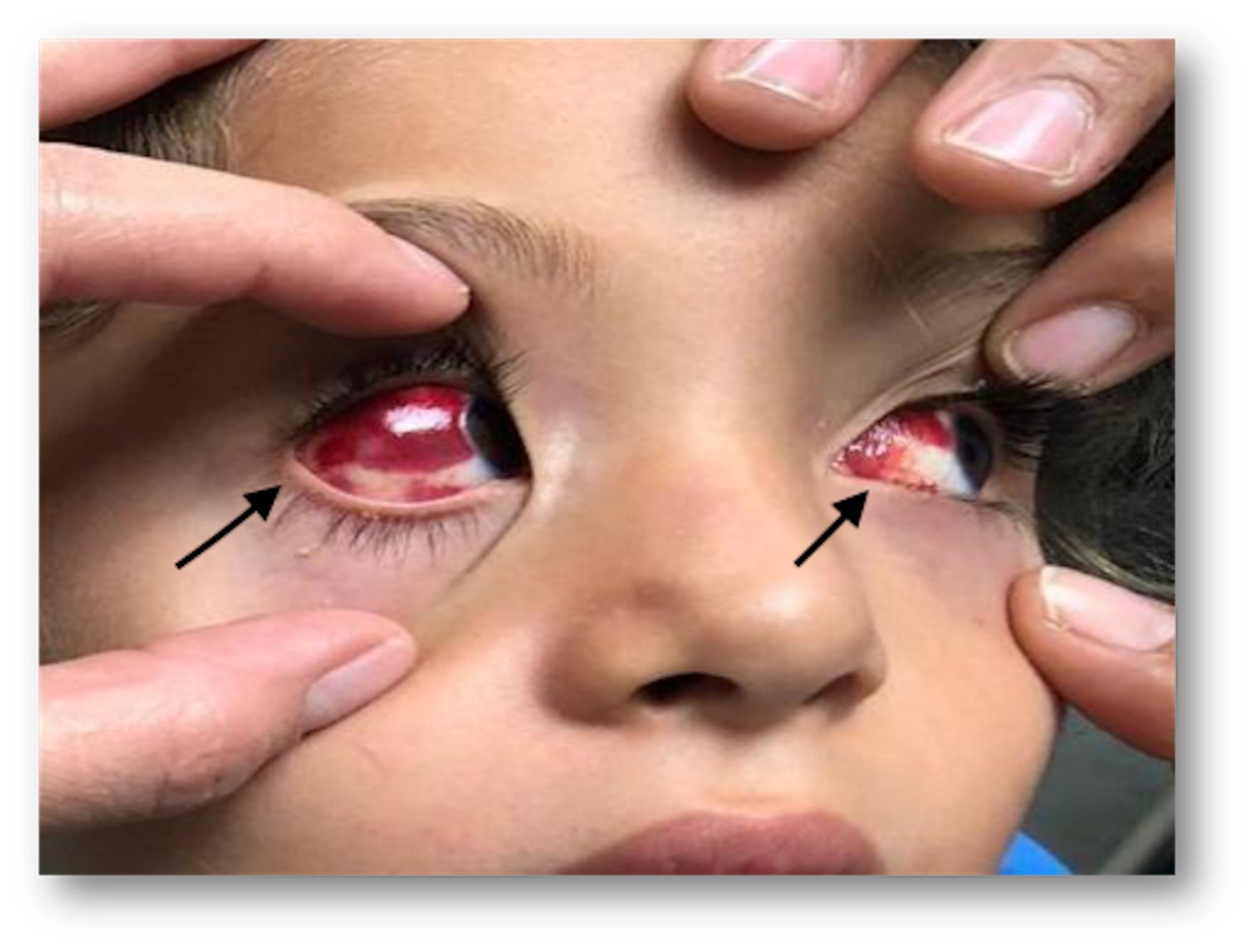
Bilateral subconjuntival hemorrhages 24 hours posterior to gammaglobulin infusion.

**Figure 3:**
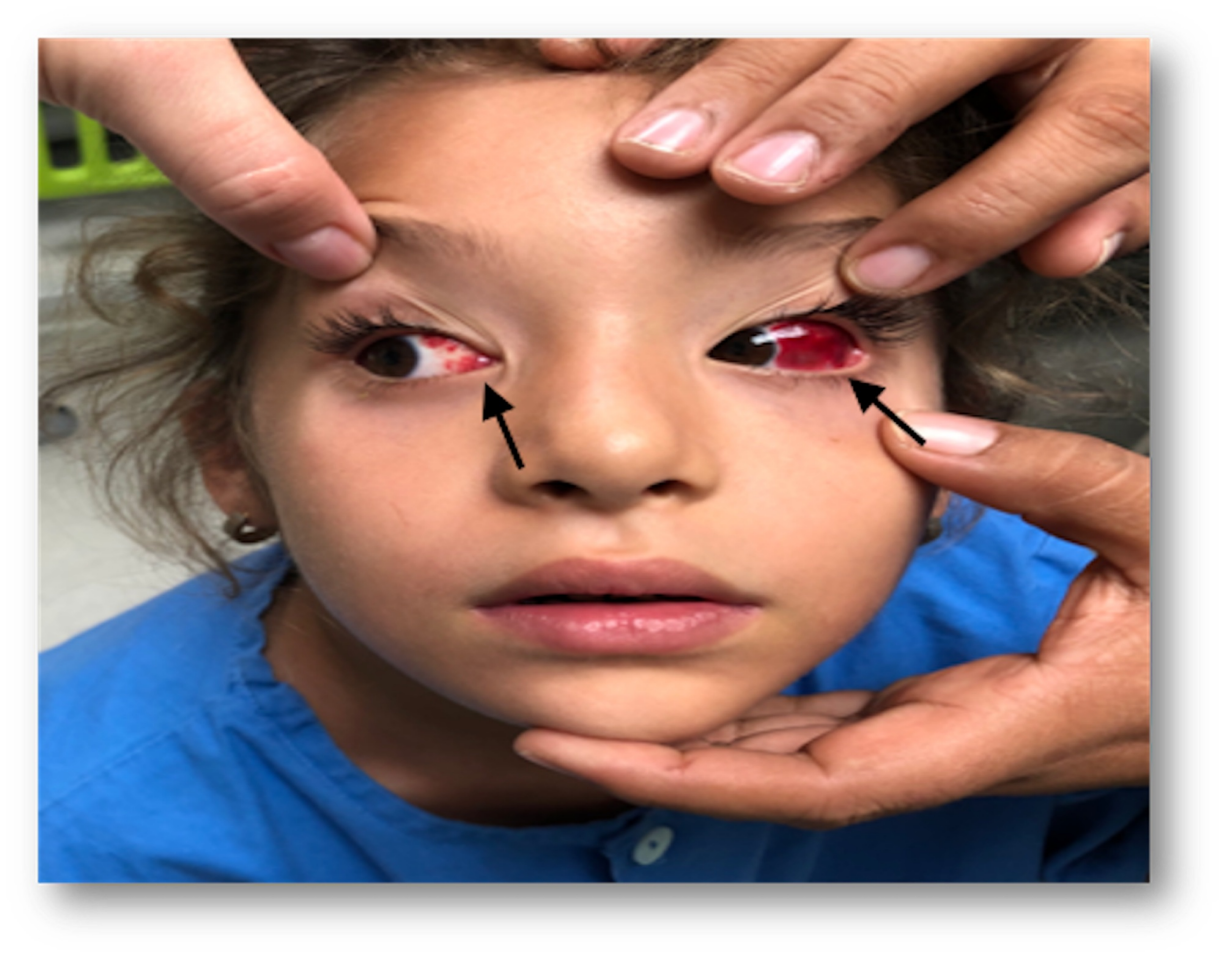
Bilateral subconjuntival hemorrhages 24 hours posterior to gammaglobulin infusion, notice greater extention of hemorrhages in the left temporal conjunctiva.

However, one day after IVIG infusion was completed, she developed extensive bilateral subconjunctival hemorrhages of temporal predominance (Figures 2, 3), which increased in intensity during the following two days especially in the left eye. Given this finding, an evaluation by pediatric ophthalmology was performed, a normal eye fundus was documented, anterior uveitis was ruled out, and conservative eye management was recommended. 

An echocardiogram during admission showed normal coronary arteries, with no abnormalities in function or structures of the heart. She was discharged on oral ASA 5 mg/kg/ day. On follow-up appointments, a second echocardiogram was normal eight weeks after. She had resolution of the hemorrhages after approximately six weeks and no visual sequelae have been documented so far. No peeling in hands or feet was described subsequently, neither thrombocytosis was found on her follow up complete blood count test. The third echocardiogram has been postponed due to the coronavirus disease 2019 (COVID-19) pandemic.

## Discussion

Kawasaki disease is a systemic vasculitis of small and medium sized vessels. KD etiology is presumed to be infectious, however, a specific agent has not been identified and the etiology is multifactorial. Complete (typical) KD diagnosis is clinical, based on the presence of fever persisting for at least five days and accompanied by four of five findings: changes in the extremities including hand and/or foot erythema or swelling during the acute stage and fingertip scaling over convalescence; polymorphous skin exanthema; bilateral conjunctivitis with no purulent discharge and classically with perilimbal sparing; oral mucositis including strawberry tongue; and cervical unilateral lymphadenopathy with a 1.5 cm or larger diameter. In some cases, patients do not fulfill the classic criteria for Kawasaki disease and are classified as having incomplete (atypical) disease, this is more common in younger infants and older children.

Ocular complications in Kawasaki disease usually occur during the acute and subacute phases, are usually transient, and resolve during the following months. The most common ocular feature is bilateral conjunctival injection, which is present in up to 90% of patients [1]. Ohno et al. in 1982 were among the first researchers to describe prospectively the ocular manifestations of KD patients, by using slit-lamp biomicroscopy, ophthalmoscopy, determination of visual acuity and tonometry whenever possible and follow them up frequently. In an analysis of 18 children, they found bilateral conjunctival injection in 16 (89%) patients, superficial punctate keratitis in 4/18 children (22%), vitreous opacities in 2/17 (12%), papilledema in 2/18 (11%), and subconjunctival hemorrhage in 1/18 (6%). Except for subconjunctival hemorrhage, these symptoms were also bilateral.

In a more recent publication of 115 KD cases from Brazil, Alves et al. described the presence of ocular complications in 13.2% of patients, being the most frequent complication anterior uveitis in 13, papilledema in one, and conjunctival hemorrhage in one, being this unilateral as well [2].

In 2007, Al-Abbadi et al. [3] performed cytopathological tests by means of Pap smear of the conjunctiva of patients with Kawasaki disease in the acute stage, and after treatment with IVIG. They found that in the acute stage of the disease there is a predominantly neutrophilic inflammatory infiltrate, which rapidly changes to mononuclear and resolves in the convalescent stage, and that similar changes occur at the peripheral blood level, which correlates with the histological changes. 

The presence of anterior uveitis in KD can manifest in up to 25 to 50% of patients, is usually self-limited, and has even been proposed as an additional diagnostic tool for early disease recognition and its timely treatment [4,5,6].

Retinal ischemia may occur as vasculitis progresses; some authors suggest prompt evaluation by an ophthalmologist if ocular complications are suspected [6]. Gao et al. in 2018 reported a case of a girl with incomplete KD and reduced eye volume, cataracts, retinal detachment, choroid, and chorioretinal folds. Besides IVIG, she was treated with eye steroids and had total recovery within one month [7]. More recently, Suganuma et al. described a seven-year-old boy who developed retinal vasculitis with impairment of visual acuity, and also refer to the only three reports in the literature with long-term visual impairment due to KD [8].

In our patient, despite the significant conjunctival affection, anterior uveitis was ruled out by ophthalmology, and this is consistent with previous reports in the literature that severe ocular complications can occur during KD in the absence of uveitis. Expectant management was given, without the need to use any other treatment than IVIG, and no topical steroids were used. 

In the vast majority of the literature, various complications of KD are described at the ocular level, both in the anterior and posterior segment, and unilateral subconjunctival hemorrhages have been described, but to the best of our knowledge, our patient represents the first case report in which bilateral subconjunctival hemorrhages manifest as a complication of KD, at least in Latin America.

## Conclusions

KD has well-defined clinical criteria, but since it is a systemic disease, unusual manifestations can occur also. The real incidence of ocular complications and in particular subconjunctival hemorrhages among children with KD is unknown, as most descriptions in the literature are single case reports, small case series, or retrospective studies. Prospective multicenter studies focusing on the ocular involvement and complications of children with KD are needed, particularly in Latin America where there is a paucity of information on this disease. 
